# Cartilage damage in patients with scapholunate lesions: arthroscopic prevalence, location and associated clinical factors

**DOI:** 10.1177/17531934251407799

**Published:** 2026-02-18

**Authors:** L van Wijk, JS Teunissen, R Feitz, Hand Wrist Study Group, EPA van der Heijden, SER Hovius

**Affiliations:** 1 Department of Plastic, Reconstructive and Hand Surgery, Radboud Institute for Health Sciences, The Netherlands; 2 Hand and Wrist Center, Xpert Clinics, The Netherlands; 3 Department of Plastic, Reconstructive and Hand Surgery, Erasmus Medical Center, The Netherlands; 4 Department of Plastic, Reconstructive and Hand Surgery, Jeroen Bosch Ziekenhuis, The Netherlands

**Keywords:** arthroscopy, cartilage, scapholunate ligament, wrist

## Abstract

**Introduction:**

Scapholunate ligament (SLL) injuries can lead to instability and osteoarthritis through stages of scapholunate dissociation and scapholunate advanced collapse. Although cartilage damage is traditionally seen as a late finding, recent evidence suggests that it may occur earlier. This study aimed to assess the prevalence and distribution of cartilage damage in patients with isolated SLL lesions confirmed by arthroscopy and to explore associated sociodemographic and clinical factors.

**Methods:**

A retrospective cohort study was conducted in patients with arthroscopically confirmed SLL lesions. Baseline demographics, injury duration, and Patient Rated Wrist Hand Evaluation (PRWHE) scores were routinely collected prior to arthroscopy. Arthroscopic findings documented cartilage damage at the scaphoid (fossa), capitate, lunate (fossa), hamate and triquetrum. Prevalence was analysed descriptively, and associations were tested using univariate logistic regression.

**Results:**

Of 226 included patients, 33.2% showed cartilage damage, most commonly affecting the scaphoid and scaphoid fossa. Damage was present across all Geissler grades, including patients without prior fractures. It was more frequent in older males, those with Geissler IV lesions, prior wrist fractures and those less likely to report a clear traumatic event. No association was found with symptom duration, dominant hand, occupation or PRWHE scores.

**Conclusion:**

The presence of cartilage damage across all Geissler grades challenges the idea that it is a late-stage finding. These results support the need for a revised classification system that integrates both ligament and cartilage pathology to enable more tailored treatment strategies for scapholunate ligament injuries.

**Level of evidence:**

Level III (Cohort study)

## Introduction

Scapholunate ligament (SLL) lesions are among the most common ligamentous wrist injuries. These lesions can disrupt wrist kinematics, progressing through defined stages of predynamic, dynamic and eventually static scapholunate dissociation (Kuo and Wolfe, 2008; Zhou et al., 2024). This sequence is traditionally associated with the development of scapholunate advanced collapse (SLAC), a predictable pattern of wrist osteoarthritis (Kuo and Wolfe, 2008; Watson and Ballet, 1984). Scapholunate advanced collapse follows a progressive course, initially involving the radioscaphoid joint (SLAC I and II), then the midcarpal joint (SLAC III) and finally total wrist osteoarthritis (SLAC IV) (Watson and Ballet, 1984; Weiss and Rodner, 2007).

Scapholunate advanced collapse is typically staged using radiological imaging. Techniques such as radiographs, computed tomography and magnetic resonance imaging have limited sensitivity and specificity in detecting the subtle cartilage changes of softening of early cartilage lesions (Adolfsson and Povlsen, 2004; Andersson et al., 2021; Dornberger et al., 2015). Wrist arthroscopy has the advantage of direct visualisation of any cartilage damage and the ability to assess the integrity of the SLL with a probe (Terzis et al., 2021).

Cartilage damage is often seen as a late consequence of scapholunate dissociation. In our previous study using arthroscopy in suspected SLL lesions, we found that cartilage damage can also be present in patients with partial scapholunate ligament tears (van Wijk et al., 2025). This observation suggests that cartilage damage occurs earlier in the disease process than previously assumed. Alternatively, both cartilage damage and SLL lesions might co-exist and be related to previous trauma or degenerative changes (Andersson et al., 2021; Lindau et al., 1997; Weiss and Rodner, 2007).

A better understanding of the prevalence and distribution of cartilage damage in SLL injuries may help to refine treatment strategies. Furthermore, identifying risk factors for cartilage damage, such as patient demographics, duration of injury, prior trauma or Geissler grade, may help predict which patients are at greater risk of progression to advanced joint disease.

The primary aim of this study was to determine the prevalence and location of cartilage damage in patients with SLL lesions confirmed via arthroscopy. The secondary aim was to evaluate potential associations between cartilage damage and sociodemographic and clinical factors.

## Methods

### Study design and setting

We conducted a retrospective cohort study using the medical records of patients who underwent diagnostic wrist arthroscopy at Xpert Clinics in The Netherlands. After obtaining informed consent, a secure online system (GemsTracker) automatically distributed questionnaires to patients (Erasmus MC, 2024). The precise setting of our cohort has been described before (Selles et al., 2020). This study used the automatically collected data for baseline characteristics and extracted additional medical history details and arthroscopic findings from the medical records. The study was conducted under the Strengthening the Reporting of Observational Studies in Epidemiology guidelines (von Elm et al., 2014). Ethical approval was obtained from our institution’s review board.

### Patients

All adult patients who underwent diagnostic wrist arthroscopy at our institution with an arthroscopically confirmed SLL lesion were included in this study. Those with previous surgical interventions involving the scapholunate ligament were excluded. Patients with concomitant injuries to other ligaments, such as the triangular fibrocartilage complex, lunotriquetral ligament or radioscaphocapitate ligament, were also excluded to avoid confounding factors in assessing cartilage damage specifically linked to SLL lesions (Palmer, 1989; Wagner et al., 2015). Patients with a history of wrist fracture were not excluded, as such fractures may be associated with both SLL lesions and cartilage damage. A subanalysis compared patients with and without a previous wrist fracture.

### Arthroscopic procedure

In our study, all wrist arthroscopies were performed by level III–IV hand surgeons (Tang and Giddins, 2016), using a 1.9 mm arthroscope (Arthrex GmbH, Utrecht). Patients were positioned supine with the elbow flexed at 90° and traction applied to the wrist. The 3–4, 4–5 or 6R portals were accessed for visualizing the radiocarpal joint, while the midcarpal ulnar and midcarpal radial portals were accessed for the midcarpal joint. The portals used in each procedure were consistently documented. Arthroscopic findings were recorded in the medical records using a standard template.

### Outcome measures

Baseline characteristics were retrieved from a regional arthroscopy database, including age, sex, duration of symptoms, type of work, dominant hand involvement and a baseline Patient Rated Wrist Hand Evaluation (PRWHE) score. Participation in questionnaires was voluntary; therefore, some data on dominant hand involvement and PRWHE scores were incomplete. Additional information, such as the location of symptoms, previous fracture and all details of the arthroscopic procedures, were obtained from medical records.

The presence of cartilage damage at specific sites was noted from standard operation reports: the scaphoid fossa, scaphoid, capitate, lunate, lunate fossa, hamate and triquetrum. The presence or absence of cartilage damage was documented but severity details were unavailable. The prevalence of cartilage damage across the different Geissler grades was recorded (Geissler et al., 1996).

To identify factors associated with cartilage damage, patients were grouped based on the presence or absence of cartilage damage in addition to an SLL lesion. The association with age, sex, dominant hand involvement, type of work, self-reported wrist trauma, history of wrist fracture, Geissler grade and baseline PRWHE scores was examined. Type of work was reported in the baseline questionnaire and divided into three categories: light (e.g. an office job), moderate (e.g. working in a shop) or heavy (e.g. working at a construction site). Self-reported wrist trauma was collected from the medical records and was scored ‘Yes’ if patients remembered falling on the wrist. History of wrist fracture was scored ‘Yes’ when patients reported having had a wrist fracture or old fractures were seen on X-rays. The validated Dutch version of the PRWHE questionnaire was sent out prior to arthroscopy to assess the patient-reported pain and hand function (AJ Videler and T Schreuders, 2008). Variables were selected based on prior research, clinical expertise and data availability.

### Analysis

Baseline characteristics were summarized with descriptive statistics. Continuous variables were presented as means with standard deviations or medians with interquartile ranges, depending on their distribution assessed with histograms and quintile–quintile plots. Categorical variables were reported as frequencies and percentages. Cartilage damage prevalence was reported in counts and percentages.

Univariate logistic regression was performed to investigate the factors associated with cartilage damage. Outcomes are presented as odds ratios (OR) with 95% confidence intervals (CI) and the corresponding *p*-value. Chi-square tests were applied to investigate differences between the groups for variables not included in the logistic regression.

## Results

**Figure 1.**
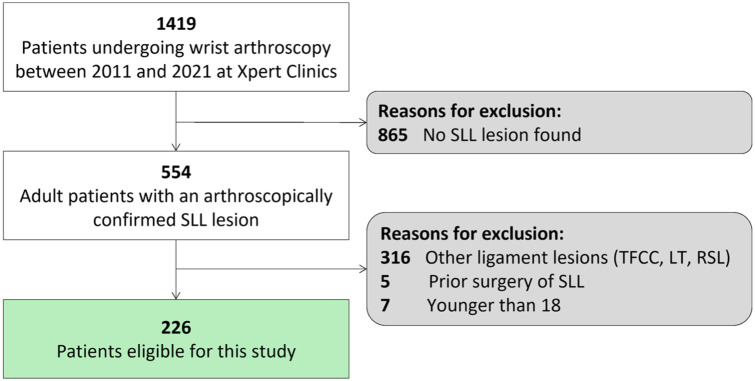
Flowchart of patient selection.

A total of 1419 patients who underwent diagnostic wrist arthroscopy at our institution were identified. Of these, 554 patients had an arthroscopically confirmed SLL lesion. After applying the exclusion criteria, 226 patients were included in this study (Figure 1).

### Prevalence of cartilage damage

**Figure 2.**
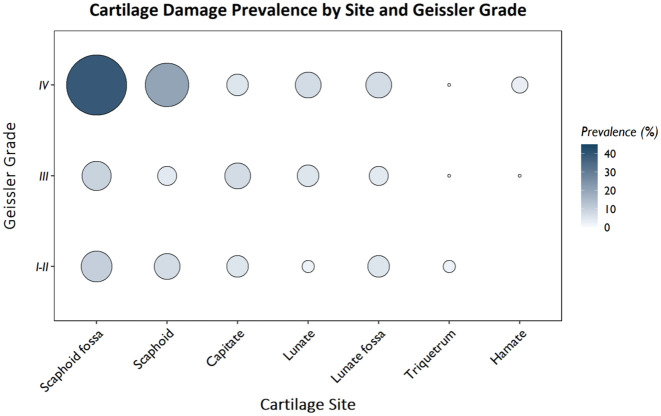
Prevalence of cartilage damage by cartilage site and Geissler grade, with the size of the circles representing the prevalence in percentages. It can be noted that cartilage damage is present across all sites and Geissler grades, but is more prevalent around the scaphoid and scaphoid fossa and Geissler grade IV.

Cartilage damage was recorded in 75 of the 226 patients (33.2%) with SLL lesions. The prevalence of cartilage damage was the highest in patients with Geissler IV lesions, with the most frequently affected areas being the scaphoid fossa and the scaphoid itself (Figure 2, Table S1). Other regions, including the capitate, lunate, lunate fossa, hamate and triquetrum, also showed cartilage changes, although no clear correlation with the Geissler classification was observed at these sites.

**Figure 3.**
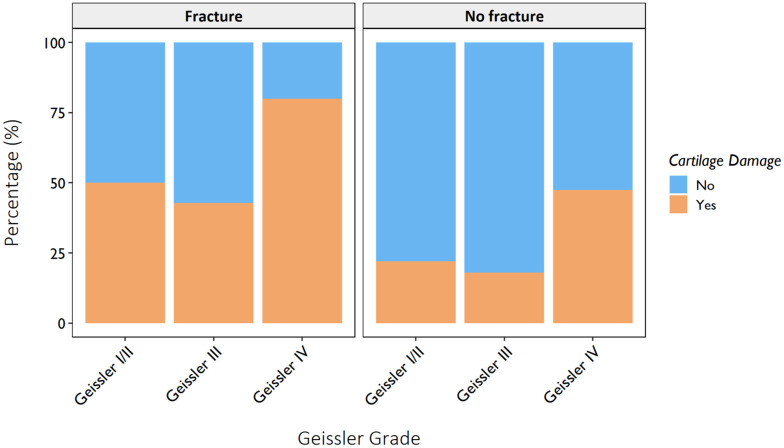
Prevalence of cartilage damage per Geissler grade and history of wrist fracture. It can be noted that cartilage damage is present across all Geissler grades, regardless of a history of wrist fracture.

**Figure 4.**
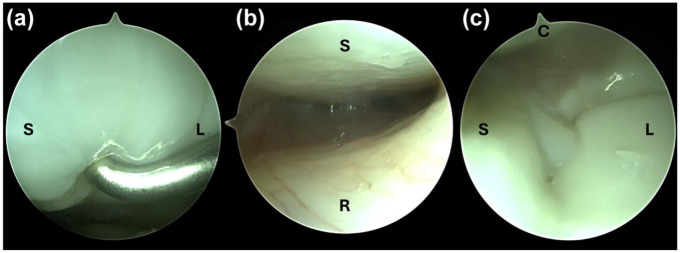
Arthroscopic images illustrating a scapholunate ligament lesion (classified as Geissler grade II) as seen from both radiocarpal and midcarpal portals. (a) Arthroscopic view of the scapholunate ligament from the 3–4 radiocarpal portal with a probe introduced via the 6R portal. The lesion was classified as Geissler grade II. (b) Arthroscopic view from the 3–4 radiocarpal portal showing slight cartilage damage of the scaphoid (S) and the scaphoid fossa of the radius (R), consistent with a so-called *kissing chondropathy*. (c) Arthroscopic image from the midcarpal space demonstrating the scaphoid (S) on the left, lunate (L) on the right and capitate (C) on top.

The prevalence of cartilage damage was higher in patients with Geissler I/II lesions compared with Geissler III lesions (Figures 2 and 3, Table S1). Many patients with Geissler I/II lesions had a history of wrist fracture (Table S1). Cartilage damage was observed across all Geissler grades independent of a history of wrist fracture (Figures 3 and 4).

### Factors associated with cartilage damage

A comparison of baseline characteristics between patients with and without cartilage damage revealed that those with cartilage damage were: male (64.0 vs. 43.7%, OR = 2.29); older (50.0 vs. 38.0 years old, OR = 1.06); with Geissler IV SLL lesions (OR = 2.59); with wrist fracture (17.3% vs. 7.3%, OR = 2.67); and with no history of traumatic wrist injury (46.7% vs 66.2%, OR = 0.45) (Table S1). Duration of symptoms, dominant hand involvement, type of work, Geissler grade III lesions and total PRWHE scores at baseline (Table S1) did not significantly differ between the groups.

## Discussion

Although not always routinely performed, wrist arthroscopy remains the reference standard for diagnosing SLL, particularly when imaging is inconclusive or symptoms persist despite normal radiographs (Andersson, 2017; De Santis et al., 2022). It can reliably grade SLL lesions, assess dynamic instability and, when partial tears are identified, facilitate immediate ligament repair in the same procedure (Geissler et al., 1996; Terzis et al., 2021; Zhou et al., 2024).

This study showed that 33.2% of patients with an arthroscopically confirmed SLL lesion also had cartilage damage. This prevalence is comparable with those in other studies of distal radial fractures and SLL lesions (17–32%) (Andersson et al., 2021; Lindau et al., 1997; Swart and Tang, 2017). Damage was most commonly observed around the scaphoid and scaphoid fossa, in keeping with established biomechanical models of SLAC staging (McLean and Taylor, 2019; Tischler et al., 2014; Watson and Ballet, 1984).

However, we also observed cartilage damage inconsistent with the SLAC progression model. Cartilage lesions were found across all Geissler grades, including early-stage (I–II) lesions, and in some patients without a history of wrist trauma or fracture. This may represent the greater sensitivity of arthroscopy to detect early changes compared with imaging techniques (Strickland et al., 2024) or indicate alternative mechanisms such as subtle post-traumatic damage, chronic microinstability from partial ligament tears or idiopathic (or age related) changes.

Although the current data cannot establish prognostic value, this observation may nonetheless be relevant, as surgical decision-making often relies on the presence and location of cartilage injury (Geissler et al., 1996; Konopka and Chim, 2018; Messina et al., 2013; Zhou et al., 2024). In current practice, cartilage damage may steer the choice towards salvage procedures, particularly when radiocarpal or midcarpal involvement is noted. Our results suggest caution with this approach, as such cartilage damage can also be present in less severe SLL injuries. Hypothetically, these patients could still benefit from reconstructive surgery rather than proceeding directly to salvage procedures, as previously described (Dullemans et al., 2024). These observations highlight the need for comprehensive assessment, considering ligament integrity, cartilage status, and the clinical context when determining the optimal surgical strategy.

The factors associated with cartilage damage, included male sex, older age, prior wrist fractures, Geissler IV lesions and those not recalling a traumatic injury. Interestingly, while degenerative osteoarthritis is often more prevalent in women (Srikanth et al., 2005), cartilage damage in SLL injuries was more frequent in men, potentially reflecting population differences (Murphy et al., 2020; Wollstein et al., 2012). Prior studies support our findings on the association between wrist fractures and cartilage damage (Caloia et al., 2008; Lindau et al., 1997; Swart and Tang, 2017), although not all fractures necessarily lead to joint degeneration (Warwick et al., 2025), also seen in this study. Patients recalling a traumatic wrist injury showed lower cartilage damage rates, presumably because they had sought medical advice and received treatment earlier.

No significant differences were found for dominant hand involvement, symptom duration, occupation or baseline PRWHE scores, suggesting that cartilage changes may progress independently of symptoms or use of the wrist (Gignac et al., (2020). Distinguishing degenerative from post-traumatic changes, and symptomatic cartilage lesions from incidental finding, is necessary to evaluate wrist pathology and guide treatment (Andersson et al., 2021).

This study has several limitations. Firstly, it relied on a database created for routine clinical practice rather than specifically for research purposes. Cartilage damage was recorded as present or absent without severity grading, restricting our analysis to a binary assessment. Preoperative data were not standardized, with some missing data, and the arthroscopic procedure itself was not standardized. While portal use was consistently documented, not all procedures had a midcarpal examination. However, portal use did not differ between patients with and without cartilage damage.

Secondly, the interpretation of arthroscopic findings was subjective, introducing potential variability (Bakker et al., 2022; Hagen et al., 2015; Löw et al., 2010). Thirdly, multiple testing in regression analyses increases the risk of false positives, and no correction methods were applied. As our analysis was limited to univariable regression, we cannot determine whether the observed associations represent independent predictors, as potential confounding between variables cannot be excluded. Lastly, the lack of follow-up data means that the clinical effects of these findings remain unknown.

In conclusion, this study demonstrates that cartilage damage is common in patients with scapholunate ligament lesions, even in early stages or without previous trauma. These findings challenge the assumption that cartilage damage is exclusively a late-stage feature of SLL injury. These results should be interpreted as descriptive epidemiological data that generate questions about the timing, mechanisms and clinical relevance of cartilage damage in SLL injury. Further longitudinal research is needed to clarify the progression of cartilage damage in SLL lesions and to establish its relevance for clinical management.

## Supplemental Material

sj-doc-1-jhs-10.1177_17531934251407799 – Supplemental material for Cartilage damage in patients with scapholunate lesions: arthroscopic prevalence, location and associated clinical factors Supplemental material, sj-doc-1-jhs-10.1177_17531934251407799 for Cartilage damage in patients with scapholunate lesions: arthroscopic prevalence, location and associated clinical factors by L. van Wijk, J.S. Teunissen, R. Feitz, E.P.A. van der Heijden and S.E.R. Hovius in Journal of Hand Surgery (European Volume)
