# Supplementary material for: Cartilage damage in patients with scapholunate lesions: arthroscopic prevalence, location and associated clinical factors
Source: J Hand Surg Eur Vol. 2026 Feb 18;51(5):597–603. doi: 10.1177/17531934251407799 (PMC13090558; doi:10.1177/17531934251407799)
Supplement: sj-doc-1-jhs-10.1177_17531934251407799 – Supplemental material for Cartilage damage in patients with scapholunate lesions: arthroscopic prevalence, location and associated clinical factors [file sj-doc-1-jhs-10.1177_17531934251407799.doc]

Table S1 Imaging parameters

| **Plane** | **Sequence** | **TR(ms)** | **TE(ms)** | **FOV(mm)** | **Section thickness (mm)** | **Gap (mm)** | **Matrix** |
| --- | --- | --- | --- | --- | --- | --- | --- |
| **Cadaveric elbow specimens** | | | | | | | |
| **Coronal** | **PD‑FS** | 4492-5392 | 45 | 150×150 | 3 | 0 | 300×265 |
| **Axial** | **PD‑FS** | 6173-8987 | 40 | 150×150 | 3 | 0 | 376×345 |
| **Sagittal** | **PD‑FS** | 5392-7489 | 40 | 150×150 | 3 | 0 | 376×318 |
| **lateral epicondylitis** | |  |  |  |  |  |  |
| **Center 1** |  |  |  |  |  |  |  |
| **Coronal** | **PD‑FS** | 3000 | 40 | 120×120 | 3 | 0.3 | 268×205 |
| **Axial** | **PD‑FS** | 3000 | 40 | 130×130 | 3 | 0.3 | 388×240 |
| **Sagittal** | **PD‑FS** | 3000 | 40 | 120×120 | 3 | 0.3 | 268×205 |
| **Coronal** | **T1WI** | 561 | 8 | 120×120 | 3 | 0.3 | 268×205 |
| **Center 2** |  |  |  |  |  |  |  |
| **Coronal** | **PD‑FS** | 3341 | 30 | 130×130 | 4 | 0.4 | 216×180 |
| **Axial** | **PD‑FS** | 3368 | 30 | 130×130 | 4 | 0.4 | 216×215 |
| **Sagittal** | **PD‑FS** | 3979 | 30 | 130×130 | 4 | 0.4 | 216×168 |
| **Coronal** | **T1WI** | 450 | 22 | 130×130 | 4 | 0.4 | 260×208 |
| **Center 3** |  |  |  |  |  |  |  |
| **Coronal** | **PD‑FS** | 2000 | 30 | 130×130 | 3 | 0.3 | 260×196 |
| **Axial** | **PD‑FS** | 2000 | 30 | 120×120 | 3 | 0.3 | 240×179 |
| **Sagittal** | **PD‑FS** | 3000 | 30 | 130×130 | 3 | 0.3 | 260×215 |
| **Coronal** | **T1WI** | 633 | 23 | 130×130 | 3 | 0.3 | 324×258 |

MRI: Magnetic resonance imaging; TR: repetition time; TE: echo time; FOV: field of view; PD‑FS: proton density-fat suppression; T1WI: T1-weighted imaging; FS: fat suppression.

**Table S2 Reproducibility analysis of some MRI parameters**

|  | **Intra‑observer reliability** | | **inter‑observer reliability** | |
| --- | --- | --- | --- | --- |
|  | **ICC** | **95%CI** | **ICC** | **95%CI** |
| **RHSP MRI Characteristics** |  |  |  |  |
| **Coronal image** |  |  |  |  |
| **Length(mm)** | **0.983** | **0.964-0.992** | **0.834** | **0.680-0.917** |
| **Thickness(mm)** | **0.989** | **0.977-0.995** | **0.821** | **0.657-0.910** |
| **Area(mm^2^)** | **0.983** | **0.965-0.992** | **0.868** | **0.741-0.935** |
| **Sagittal image** |  |  |  |  |
| **Length(mm)** | **0.955** | **0.908-0.979** | **0.890** | **0.782-0.946** |
| **Thickness(mm)** | **0.913** | **0.826-0.958** | **0.836** | **0.685-0.919** |
| **Area(mm^2^)** | **0.988** | **0.975-0.994** | **0.875** | **0.754-0.938** |
| **Size of CET tear (mm)** | **0.994** | **0.987-0.997** | **0.823** | **0.661-0.912** |
| **Grade of LCL complex abnormality** | **0.952** | **0.902-0.977** | **0.812** | **0.642-0.906** |
| **Muscle oedema, n (%)** | **0.935** | **0.869-0.969** | **0.800** | **0.621-0.900** |

MRI: Magnetic resonance imaging; RHSP: radiohumeral synovial plica; CET: common extensor tendon; LCL lateral collateral ligament.
